# P-SILI in critically ill COVID-19 patients: Macklin effect and the choice of noninvasive ventilatory support type

**DOI:** 10.1186/s13054-023-04313-z

**Published:** 2023-01-24

**Authors:** Alessandro Belletti, Luigi Vetrugno, Cristian Deana, Diego Palumbo, Salvatore M. Maggiore, Giovanni Landoni

**Affiliations:** 1 Department of Anesthesia and Intensive Care, IRCCS San Raffaele Scientific Institute, Via Olgettina 60, 20132 Milan, Italy; 2 Department of Medical, Oral and Biotechnological Sciences, Gabriele d’Annunzio University of Chieti Pescara, Chieti, Italy; 3 Anesthesia and Intensive Care 1, Department of Anesthesia and Intensive Care, Health Integrated Agency Friuli Centrale, Udine, Italy; 4 Department of Radiology, IRCCS San Raffaele Scientific Institute, Milan, Italy; 5 Department of Innovative Technologies in Medicine and Dentistry, Gabriele d’Annunzio University of Chieti Pescara, Chieti, Italy; 6 School of Medicine, Vita-Salute San Raffaele University, Milan, Italy

We read with great interest the article by Elabbadi et al. [1] about the role of spontaneous pneumomediastinum (PMD) as a marker of patient self-induced lung injury (P-SILI) in patients with coronavirus disease 2019 (COVID-19) acute respiratory distress syndrome (ARDS).

It is recognized that excessive inspiratory effort and high transpulmonary pressures during patient spontaneous breathing may lead to P-SILI and barotrauma.

In their article, the authors hypothesized that the Macklin effect could be a pathophysiological explanation for the development of spontaneous PMD in COVID-19 patients not receiving invasive mechanical ventilation (IMV).

We have two comments on this study.

First, we would like to underline that the Macklin effect (also known as Macklin-like radiological sign or interstitial emphysema) is generally detectable on chest computed tomography (CT) scan as a linear collection of air tracking along with bronchovascular bundles, visceral pleura and/or interlobular septa [2]. Our group recently demonstrated that the Macklin effect (detected on chest CT scan) is an accurate predictor of barotrauma development (PMD and/or pneumothorax) in patients with COVID-19 ARDS (sensitivity: 89.2% [95% CI 74.6–96.9]; specificity: 95.6% [95% CI 90.6–98.4]) and is detectable 8–12 days before clinically overt barotrauma [2]. We also found a relationship between the topographical distribution of Macklin effect within the lung parenchyma and the temporal interval to the first radiological evidence of barotrauma: the smaller the bronchovascular sheath involved, the longer the temporal advance [2].

Accordingly, as Macklin effect precedes overt PMD development by several days, we can now hypothesize that early identification of Macklin effect could be used to identify patients at risk for or with ongoing P-SILI and select patients for different treatment algorithms.

Second, the authors did not comment on the type of noninvasive ventilatory support and the patient clinical condition. However, as recently reported by our group, continuous positive airway pressure/pressure-support ventilation (C-PAP/PSV), compared with conventional oxygen therapy (COT), increased the risk of barotrauma, while high-flow nasal oxygen (HFNO) did not [3].

Of note, patients that underwent only COT, only HFNO, only C-PAP/PSV, only IMV versus escalation therapies COT/HFNO, COT/HFNO/C-PAP/PSV, COT/HFNO/PSV/IMV presented fewer barotrauma events (*p* < 0.001) [3]. This may suggest that when the first noninvasive respiratory support fails, it could be better to abolish the patient respiratory effort and to start directly invasive mechanical ventilation instead of trying different forms of noninvasive respiratory support, at least in the more severe hypoxemic patients.

Furthermore, these findings reinforce the concept that excessive transpulmonary pressure such as those developed during inspiration supported by PSV in patients with respiratory distress may exacerbate P-SILI and may indeed induce barotrauma through the Macklin effect.

Therefore, the choice of adequate noninvasive ventilatory support could become fundamental to mitigate Macklin's progression. In this view, personalizing the respiratory support based on the intensity or respiratory effort may be a reasonable approach.

However, we acknowledge that finding the best management strategy for patients with worsening respiratory failure and at high risk for P-SILI and barotrauma (for example, presence of Macklin effect on chest CT scan) remains to be determined.

As a matter of fact, considering the aforementioned limitation, we suggest using advanced respiratory monitoring (electrical activity of the diaphragm, lung ultrasound, electrical impedance tomography, esophageal pressure [4]) to assess the risk of P-SILI early. Patients at high risk for P-SILI and barotrauma could be candidates for strategies to mitigate this risk, for example, decision to proceed to early intubation and institution of invasive protective mechanical ventilation. In severe cases, early extracorporeal support together with ultraprotective ventilation could be an alternative approach, even in patients not meeting the “conventional” gas exchange criteria for extracorporeal support.

An interesting, alternative approach could be early use of extracorporeal support without invasive ventilation [5]. In a recently published case series, seven patients with severe COVID-19 ARDS not yet receiving IMV and with evidence of Macklin effect underwent awake implantation of veno-venous extracorporeal membrane oxygenation without IMV (patients received HFNC or NIV). No patient developed barotrauma, and five survived to hospital discharge [5].

Unfortunately, management of patients with ARDS not receiving IMV remains poorly investigated. We believe that future studies should investigate whether is possible to combine advanced monitoring tools, radiologic and clinical findings to identify the patients at risk for P-SILI and identify the optimal respiratory support as well as optimal timing for escalating therapy.

## Data Availability

Not applicable—This letter does not report original data.
